# Functionalized mesoporous bioactive glass scaffolds for enhanced bone tissue regeneration

**DOI:** 10.1038/srep19361

**Published:** 2016-01-14

**Authors:** Xingdi Zhang, Deliang Zeng, Nan Li, Jin Wen, Xinquan Jiang, Changsheng Liu, Yongsheng Li

**Affiliations:** 1 Lab of Low-Dimensional Materials Chemistry, Key Laboratory for Ultrafine Materials of Ministry of Education, School of Materials Science and Engineering, East China University of Science and Technology, Shanghai 200237, China; 2 Department of Prosthodontics, Ninth People’s Hospital affiliated to Shanghai Jiao Tong University, School of Medicine, Shanghai 200011, China; 3 Engineering Research Center for Biomedical Materials of Ministry of Education, East China University of Science and Technology, Shanghai 200237, China

## Abstract

Mesoporous bioactive glass (MBG), which possesses excellent bioactivity, biocompatibility and osteoconductivity, has played an important role in bone tissue regeneration. However, it is difficult to prepare MBG scaffolds with high compressive strength for applications in bone regeneration; this difficulty has greatly hindered its development and use. To solve this problem, a simple powder processing technique has been successfully developed to fabricate a novel type of MBG scaffold (MBGS). Furthermore, amino or carboxylic groups could be successfully grafted onto MBGSs (denoted as N-MBGS and C-MBGS, respectively) through a post-grafting process. It was revealed that both MBGS and the functionalized MBGSs could significantly promote the proliferation and osteogenic differentiation of bMSCs. Due to its positively charged surface, N-MBGS presented the highest *in vitro* osteogenic capability of the three samples. Moreover, *in vivo* testing results demonstrated that N-MBGS could promote higher levels of bone regeneration compared with MBGS and C-MBGS. In addition to its surface characteristics, it is believed that the decreased degradation rate of N-MBGS plays a vital role in promoting bone regeneration. These findings indicate that MBGSs are promising materials with potential practical applications in bone regeneration, which can be successfully fabricated by combining a powder processing technique and post-grafting process.

In recent years, the increasing number of bone tissue defects in surgery has driven the urgent need for biomaterials for applications in bone tissue regeneration^1^. Of the various candidates, mesoporous bioactive glass (MBG) has attracted much attention because of its bioactivity, biocompatibility, and osteoconductive properties. MBG possesses a similar composition to traditional bioactive glass (SiO_2_-CaO-P_2_O_5_) but exhibits higher bioactivity due to its highly ordered mesoporous structure, large specific surface area and high pore volume^2^,^3^,^4^. Shorter reaction times are needed to form hydroxycarbonate apatite (HCA) layers on the surface of MBG that have the capability of bonding with living bone after soaking in simulated body fluids (SBF)^5^. It has also been verified that Ca and Si can stimulate osteoblast proliferation and differentiation^6^. Moreover, the ordered mesoporous channels of MBG are capable of loading large amounts of drugs^7^,^8^,^9^,^10^. Thus, MBG has presented great potential for applications in bone regeneration.

Bone possesses a hierarchically porous structure ranging from 20 to 400 μm, which is necessary for bone cells to penetrate, adhere, grow and proliferate to form new bone^11^. To efficiently regenerate bone tissue and correct defects, implants should mimic this hierarchical structure. Consequently, powders cannot act as effective implants and must be built into three-dimensional porous scaffolds consisting of an interconnected macroporous network with pore diameters of at least 100 μm^12^,^13^. Thus far, the common route for preparing MBG scaffolds is through use of the polyurethane foam template method, which is achieved by combining polyurethane foam and evaporation-induced self-assembly (EISA)^14^,^15^. This type of MBG scaffold possesses an interconnected porous structure but has very low mechanical strength (0.06 MPa), which makes it difficult to use for *in vivo* bone regeneration. Based on previous studies, MBG has shown possibility to be an excellent candidate for use in bone regeneration, but its development has been greatly limited due to the severe lack of investigations *in vivo*^16^,^17^,^18^,^19^,^20^. Therefore, preparing MBG scaffolds with sufficient mechanical strength remains a major challenge.

Surface characteristics are vital for biomaterials and are usually modified by grafting organic functional groups; such modifications can greatly improve the interaction performance between biomaterials and cells^21^. For instance, different functional groups can influence cell adhesion by changing the hydrophilicity-hydrophobicity of surfaces^22^,^23^. The capability of a material to adsorb protein is also related to its functional groups, which can correspondingly influence cell adhesion^24^,^25^. Keselowsky *et al.* demonstrated that -NH_2_ possessed the strongest capability to adsorb fibronectin (FN) compared with -CH_3_, -COOH and -OH^26^. In addition to cell adhesion, functional groups can also improve stem cell differentiation. It has been shown that -NH_2_ could induce the osteogenic differentiation of human mesenchymal stem cells (hMSCs)^27^,^28^,^29^. Due to the abundance of Si-OH groups on the surface, silica-based mesoporous materials can easily be functionalized through a post-grafting process, which is usually employed to enhance the drug loading capacities and control the release rate of the loaded drugs^30^,^31^,^32^. Nevertheless, in addition to difficulties in the preparation of such scaffolds, MBG has the major disadvantage of having high degradation rates resulting from large specific surface areas and high pore volumes. Biodegradability is important when materials are used for bone tissue scaffolds; however, high degradation rates negatively affect cell attachment and growth and consequently hinder the promotion of new bone formation^33^. It is well known that the specific surface area, pore volume, and most importantly, surface characteristics of materials can be partially decreased or altered due to the grafting of functional groups. Therefore, it has been postulated that the high degradation rates of MBG could be partially decreased, while maintaining bioactivity, by introducing organic functional groups.

In this paper, a novel type of MBG scaffold (MBGS) with high mechanical strength was fabricated using a simple powder processing technique, and amino or carboxylic groups were grafted onto MBGSs through post-grafting processes. Then, the *in vitro* and *in vivo* osteogenetic capabilities of the MBGS and functionalized MBGSs were investigated comprehensively. Rabbit bone marrow stromal cells (bMSCs), which have been widely used to evaluate osteogenic differentiation, were chosen to determine the *in vitro* performance of scaffolds. The effects of the scaffolds on the proliferation and osteogenic differentiation of bMSCs were evaluated by MTT analysis, an alkaline phosphatase (ALP) activity assay, and real-time PCR analysis of osteogenic genes. A rabbit femur defect model was utilized to assess the osteogenetic capabilities of the scaffolds *in vivo*.

## Results

### Characterization of MBGSs

**Figure 1:**
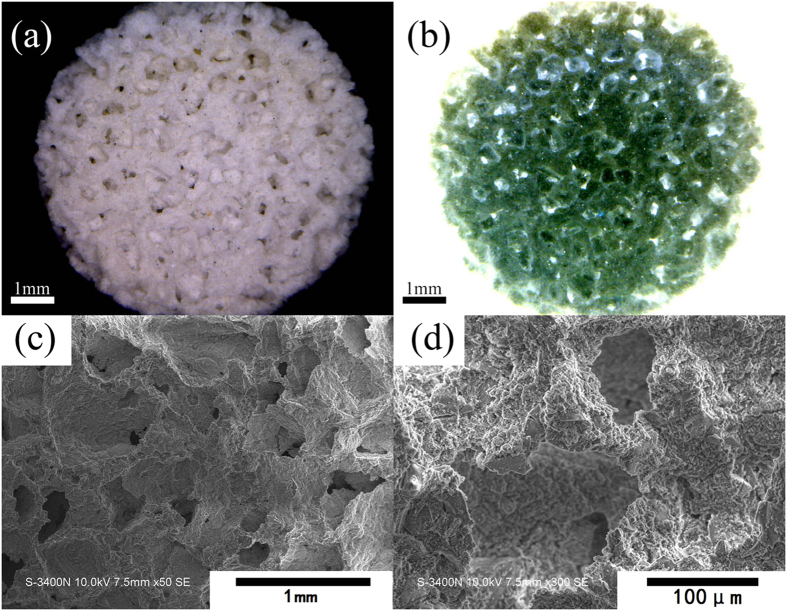
(**a**) Digital microscopic photograph, (**b**) reverse colour photograph, (**c**) SEM image and (**d**) SEM image in high magnification of the MBGS.

Figure S1 presents the photographs of the MBGS samples. It was found that MBGSs with different sizes and shapes were successfully constructed with a simple powder processing technique. Moreover, the interconnected macrostructure could be clearly seen in digital microscopic photographs (Fig. 1a,b). The SEM images shown in Fig. 1c,d further demonstrate the interconnected macrostructure. The macroporous diameter was approximately 100–300 μm (Fig. S2). Table 1 lists the compressive strengths and porosities of the MBGSs and the control sample. The compressive strength of the MBGS was approximately 0.30 Mpa and was higher than that of the contrast sample. Additionally, the porosity of the MBGS was comparable to the control sample.

**Figure 2:**
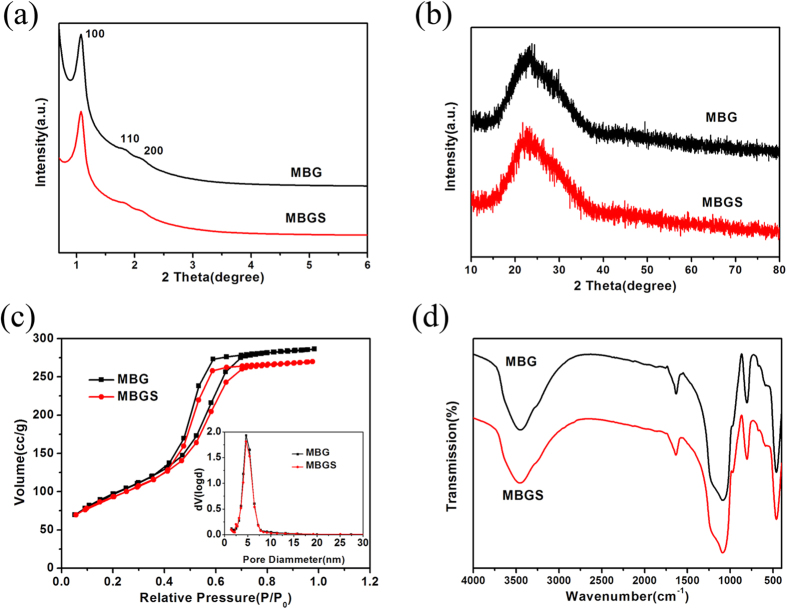
(**a**) Small-angle XRD patterns, (**b**) wide-angle XRD patterns, (**c**) nitrogen adsorption-desorption isotherms (inset: the pore size distribution curves) and (**d**) FT-IR spectra of MBG and the MBGS.

Figure 2a shows the small-angle XRD patterns of MBG and the MBGS. Both of them revealed three diffraction peaks in the small-angle regime, which were indexed to the (100), (110) and (200) diffraction peaks. These peaks indicated that both MBG and the MBGS possessed highly ordered 2D-hexagonal mesoporous structures. Wide-angle XRD patterns were collected to determine the amorphous states of the samples. As shown in Fig. 2b, only one broad diffraction peak at 2θ = 15–35° was observed for MBG and the MBGS, revealing their amorphous states. The mesoporous structures of MBG and the MBGS were investigated by N_2_ adsorption-desorption analyses. As shown in Fig. 2c, the isotherms of MBG and the MBGS were revealed to be type IV curves, and their pore sizes were approximately 4.6 nm with narrow distributions. Table 2 shows that the specific surface areas, the pore volumes and the pore sizes of MBG and the MBGS were similar. These observations demonstrated that the powder processing technique did not affect the ordered mesoporous structure of MBG. Combining this finding with the results obtained by Mercury porosimetry (Fig. S2), it was concluded that hierarchically porous MBGSs with both macropores and mesopores were successfully fabricated.

An FT-IR spectrometer was employed to measure the residuals of PVP and PEG in the MBGS. As shown in Fig. 2d, no characteristic peaks of PVP and PEG were detected, indicating the complete removal of PVP and PEG during the high-temperature calcination process.

### Characterization of functionalized MBGSs

**Figure 3:**
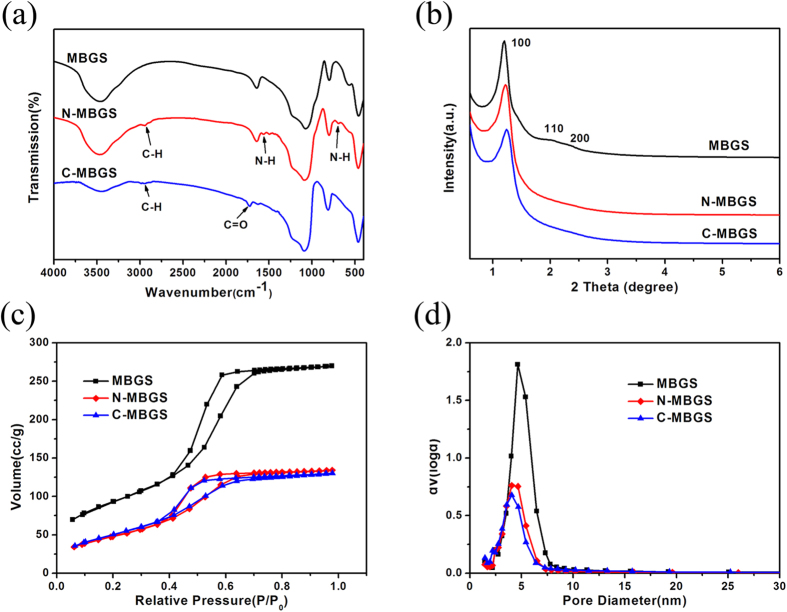
(**a**) FT-IR spectra, (**b**) small-angle XRD patterns, (**c**) nitrogen adsorption-desorption isotherms and (**d**) pore size distribution curves of the MBGS, N-MBGS and C-MBGS.

Figure 3a illustrates the FT-IR spectra for MBGS, N-MBGS and C-MBGS. Compared to the MBGS, the N-MBGS exhibited characteristic absorption peaks of N–H bonds at 1563 cm^−1^ and 689 cm^−1^, as well as the vibration of C–H bonds at 2936 cm^−1^. For the C-MBGS, absorption bands were observed at 1718 cm^−1^ and 2936 cm^−1^, which were attributed to the vibration of C = O bonds and C–H bonds, respectively. These observations demonstrated that amino and carboxyl groups were successfully grafted onto the MBGS.

Figure 3b shows the small-angle XRD patterns of MBG and functionalized MBGS samples. In contrast to MBG, the N-MBGS and C-MBGS showed only one (100) diffraction peak in the small angle region, indicating that the ordered mesoporous structure of MBG was partially destroyed during the process of grafting functional groups. As shown in Fig. 3c, it was found that the maximum adsorption capacities of both the N-MBGS and C-MBGS decreased after the grafting of functional groups, although the isotherms were still type-IV isotherms. Accordingly, the specific surface areas, pore volumes and pore sizes of the functionalized MBGSs were smaller than those of the unfunctionalized MBGS (Table 2). These observations further verified that the MBGS was successfully modified with amino or carboxyl groups.

There was no distinct difference detected in the morphologies and macroporous diameters of the MBGS and functionalized MBGS samples (Figs S2 and S3). However, the compressive strengths of the N-MBGS (0.34 ± 0.02 MPa) and C-MBGS (0.35 ± 0.02 MPa) were slightly increased with grafting of the functional groups (Table 1), demonstrating that the post-grafting process was an effective tool for modifying the hierarchical MBGS.

### Ion release, degradation and bioactivity properties of the MBGS and functionalized MBGSs *in vitro*

Bioactivity is one of the most important characteristics of MBG for bone regeneration, which can be confirmed by monitoring the growth of HCA after immersing MBG in SBF for defined periods of time. As shown in Fig. S4a, typical vibrational bands of HCA were detected; the peaks at 1487, 1417 and 873 cm^−1^ were assigned to the C–O vibration bands, and the peaks at 1040, 963, 606 and 568 cm^−1^ were assigned to the crystalline P–O bands. Figure S4b presents the wide-angle XRD patterns of scaffolds soaked in SBF for 24 h. Compared with the samples that were not soaked in SBF, all three samples exhibited new diffraction peaks that were characteristic of the apatite phase (Standard card number: JCPD 24–0033). These observations verified that all the scaffold samples were bioactive, and especially, the powder and post-grafting processes did not affect their bioactivities.

Figure S5 shows the changes of Ca, Si, and P ion concentrations in SBF with various soaking periods for the scaffold samples. The Ca ion concentrations for all the scaffolds increased significantly in the first 2 h then gradually decreased and stabilized after 48 h. Changes in P ion concentrations presented a similar trend to Ca ions. It is reasonable that the concentrations of Ca and P ions increased upon their release from the scaffolds in the initial stage and decreased with the formation of HCA because of the consumption of Ca and P ions in SBF. These observations verified that HCA could rapidly form after the soaking of various scaffolds in SBF, which is another expression of bioactivity *in vitro*. As Si ions did not contribute to the formation of HCA, the concentration of Si ions gradually increased and reached a maximum after 8 h (Fig. S5c).

Weight loss in a buffer solution is usually used to assess the degradation rate of scaffolds *in vitro*^34^,^35^. PBS (pH = 7.4) was chosen for this assay because of the formation of HCA in SBF, which makes it inconvenient to calculate weight loss. As shown in Fig. S5d, all scaffolds demonstrated weight loss with prolonged soaking periods, and the weight losses of the MBGS, N-MBGS and C-MBGS at 10 d were approximately 10%, 6% and 6%, respectively. These observations demonstrated that the degradation rate of the MBGS *in vitro* was higher than that of the functionalized MBGSs, which was probably caused by the partial decreases in specific surface area and pore volume after grafting organic functional groups into the mesoporous channels.

### Attachment, proliferation and ALP activity of bMSCs on the MBGS and functionalized MBGSs

**Figure 4:**
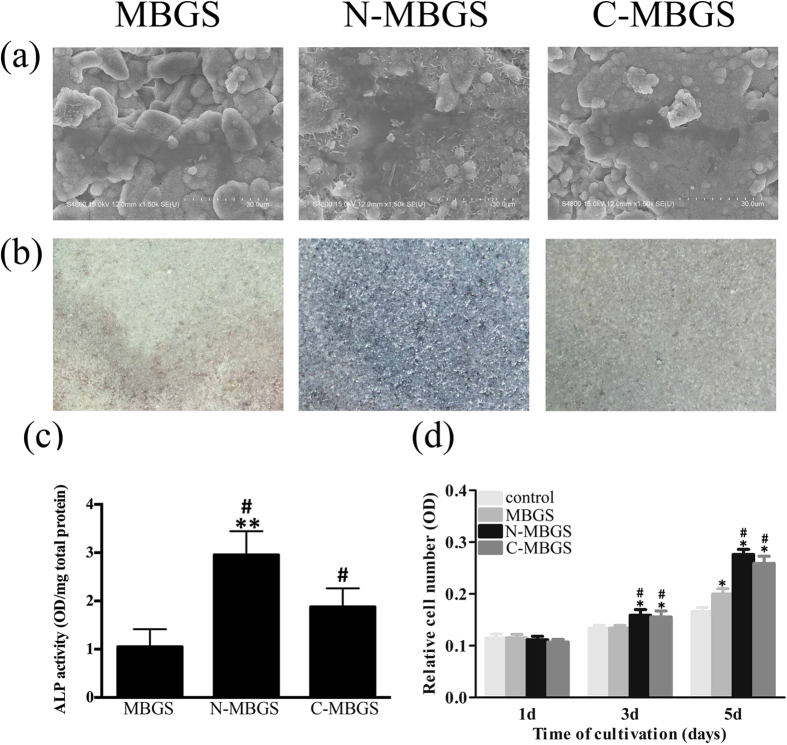
(**a**) SEM images of bMSCs on different scaffolds after culturing for 24 h, (**b**) ALP staining photographs of bMSCs cultured on different scaffolds, (**c**) semi-quantitative assay analysis of ALP activity, and (**d**) the proliferation of bMSCs cultured with MBGS, N-MBGS and C-MBGS in osteogenic medium at days 1, 3 and 5d. *P < 0.05 vs. control; ^#^P < 0.05 vs. MBGS; **P < 0.05 vs. C-MBGS.

Figure 4a shows SEM images of bMSCs grown on scaffolds. It can be observed that the bMSCs spread well and partially covered the surface of the MBGS and functionalized MBGSs. The proliferation of the bMSCs cultured with the scaffolds was detected using an MTT assay. As shown in Fig. 4d, the bMSCs proliferated over the entire culture period on all three scaffolds. On day 1, there were no significant differences between the control and the samples. By day 3, the number of cells on the MBGS was similar to that of the control; however, more cells were measured on the functionalized MBGSs. With the culture period extended to day 5, many more cells were counted on all three scaffolds, especially on the N-MBGS, compared with the control. These observations indicated that the N-MBGS presented the most potent proliferative effect for bMSCs.

ALP activity, which is an early marker of bMSC differentiation, was measured to evaluate the osteogenic differentiation of bMSCs cultured with scaffolds. From the results presented in Fig. 4b, it is obvious that the most intensive ALP staining was detected on the N-MBGS after 7 d of culture. The semi-quantitative data are consistent with the staining results. In particular, the ALP activity of the N-MBGS group was more than double the activity of the MBGS group (Fig. 4c).

### Osteogenic gene expression for bMSCs on the MBGS and functionalized MBGSs

**Figure 5:**
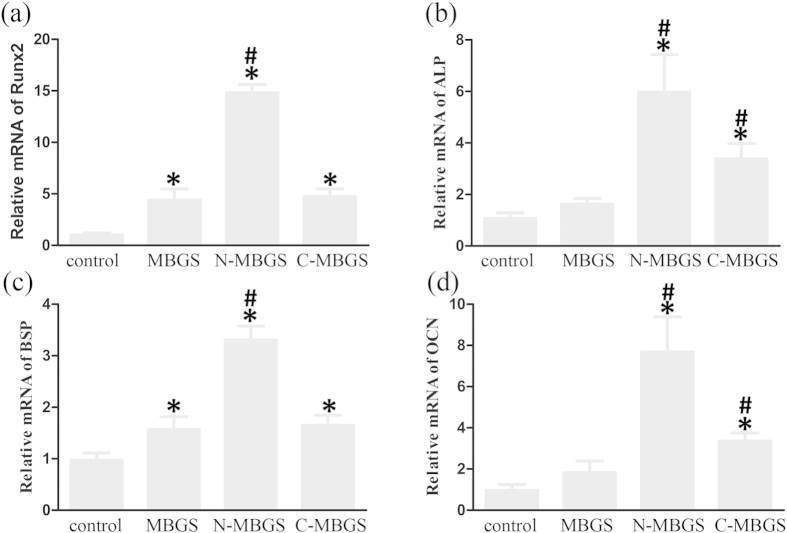
The osteogenic gene expression of (**a**) Runx2, (**b**) ALP, (**c**) BSP and (**d**) OCN for bMSCs cultured with MBGS, N-MBGS and C-MBGS after 7 days, as determined by real-time PCR analysis. *P < 0.05 vs. control; ^#^P < 0.05 vs. MBGS.

To further evaluate the osteogenic differentiation of bMSCs, the osteogenic gene expression of runt-related transcription factor 2 (Runx2), ALP, bone sialoprotein (BSP), and osteocalcin (OCN) was monitored by real-time PCR after culturing bMSCs on the three scaffolds for 7 d. As shown in Fig. 5, the expression of Runx2, ALP, BSP, and OCN was enhanced for the bMSCs on all three scaffolds, in contrast to the control. The expression of Runx2 and BSP were similar for the bMSCs on the MBGS and C-MBGS, while the expression of ALP and OCN was stronger for the bMSCs on the C-MBGS than for those on the MBGS. Notably, the expression of all osteogenic genes was significantly enhanced for the bMSCs on the N-MBGS. These observations, which were in agreement with the results of the ALP activity assay, verified that the N-MBGS possessed the highest *in vitro* osteogenic capability of the three scaffolds.

### Bone regeneration utilizing the MBGS and functionalized MBGSs in rabbit femoral defects

**Figure 6:**
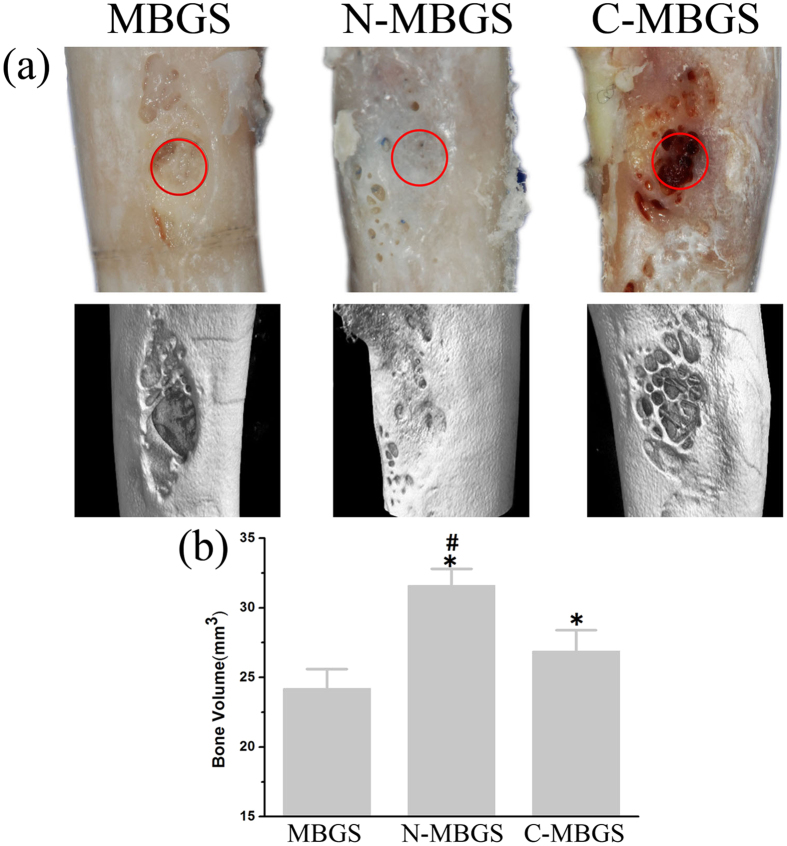
Bone regeneration after implanting the MBGS, N-MBGS and C-MBGS in rabbit femur defects for 12 weeks. (**a**) Photos (first row) and 3D images from micro-CT scans (second row) of rabbit femurs implanted with the MBGS, N-MBGS and C-MBGS (the defects are in the red circles). (**b**) Quantitative analysis of the new bone volume after 8 weeks of surgery, as determined by micro-CT. *P < 0.05 vs. MBGS; ^#^P < 0.05 vs. C-MBGS.

Micro-CT analyses were conducted to evaluate the *in vivo* osteogenic capabilities of the MBGS and functionalized MBGSs after implanting the scaffolds in rabbit femur defects for 12 weeks. Figure 6a shows photographs and 3D images from the micro-CT scans of rabbit femurs implanted with the MBGS and functionalized MBGSs. The defects can be obviously observed in the rabbit femurs implanted with the MBGS and C-MBGS; however, they could hardly be seen in the femurs implanted with the N-MBGS. The new bone volumes within the defects were calculated and are shown in Fig. 6b. The highest bone volume was generated in the defect implanted with the N-MBGS, which was consistent with observations from the images.

**Figure 7:**
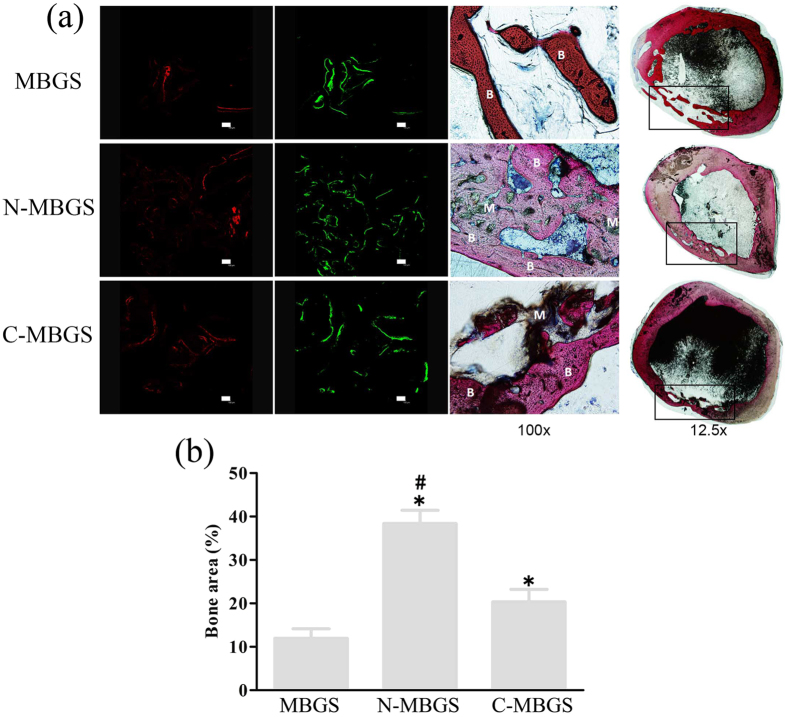
(**a**) Histological observation of new bone formation in the three types of scaffolds after 12 weeks. The colour images represent sequential fluorescent labelling of alizarin red (AL, red) and calcein (CA, green) (bar :100 μm); staining image, Left: the corresponding high magnification images of the defect sites (magnification:100×), Right: cross sections of implanted rabbit femurs (magnification :12.5×) (defects are in the black rectangles; B: new bone, M: materials). (**b**) Quantitative analysis of new bone areas after 12 weeks determined by histological observations. *P < 0.05 vs. MBGS; ^#^P < 0.05 vs. C-MBGS.

To assess the osteogenic capabilities of the scaffolds in detail, histological analyses were performed to observe the formation of new bone. Figure 7a presents the percentage of staining with each fluorochrome, the photomicrographs of the cross sections of rabbit femurs and the corresponding high magnification images of defect sites. It was clear that the amount of new bone in the defect site implanted with the N-MBGS was much higher than those implanted with the other scaffolds. Quantitatively, the new bone area of the N-MBGS group was 38% ± 5.26%, which was much higher than the areas of 20.37% ± 5.01% quantified in the C-MBGS and 12% ± 3.74% quantified in the MBGS group (Fig. 7b). This finding further verified that the *in vivo* osteogenic capability of the MBGS was significantly enhanced with the grafting of amino groups. At 4 (red) and 8 (green) weeks after operation, the percentage of the fluorochrome in the N-MBGS group was more than the percentages observed for the MBGS and C-MBGS groups. Moreover, residual materials could be observed in the higher magnification images of the defect sites implanted with the N-MBGS and C-MBGS, but not for the MBGS. These observations demonstrated that the high degradation rate of the MBGS was effectively controlled with the introduction of functional groups, in accordance with the results from the *in vitro* degradation assay.

## Discussion

In terms of the scaffolds and their effects on bone tissue regeneration, it is important to provide high mechanical stability and an appropriate degradation rate for cell penetration, adhesion, growth and proliferation for the formation of new bone. As the compressive strength of spongy bone is in the range of 0.2–4 MPa, it is necessary that the compressive strength of the designed scaffolds fall within this range^36^. It is known that the compressive strength of MBGSs prepared with a polyurethane foam template method is as low as 0.06 MPa^33^, which makes it unsuitable for *in vivo* applications in bone regeneration. However, the compressive strength of the MBGS prepared in this work was as high as 0.3 MPa with over 70% porosity (Table 1). This indicates that the simple powder processing technique is sufficient for constructing hierarchical porous scaffolds. Furthermore, it was demonstrated that MBGSs with varied shapes and sizes could be successfully fabricated using this approach, which is necessary for this technique to be applied in treating bone defects of different shapes and sizes. It is known that binding agents play a crucial role in the fabrication process, and liquid binders are usually employed to fulfil this task^37^,^38^. However, this method is problematic because liquid binders are adsorbed into the abundant mesopores of MBG, which could possibly weaken the binding capability and destroy the mesostructure of MBG. Additionally, the toxicity that results from the introduction of binders in the preparation or removal process is another issue that must be considered. PVP, which has been approved for many uses by the U.S. Food and Drug Administration (FDA), is widely used as a binder in many pharmaceutical tablets^39^. Therefore, PVP was employed as solid binder in this work. Furthermore, it was verified that PVP could be removed completely during the high-temperature calcination process (Fig. 2d).

Post-grafting is commonly used to modify silica-based mesoporous materials based on the facile reaction between the abundant Si-OH groups on the surface of the materials and organic silanes. In this work, due to the successful grafting of functional groups on the MBGS, especially onto the outer surface of the mesopores, the specific surface areas, pore volumes and pore sizes of the functionalized MBGS samples were thus decreased (Table 2). Interestingly, the release rate of Si ions from the functionalized MBGS was found to be slower than that of the MBGS, which induced the decrease in the degradation rate of the MBGS (Fig. S4c,d). More importantly, the compressive strength was found to slightly increase with the introduction of functional groups and the corresponding partial collapse of the mesostructures. Nevertheless, the bioactivities of the functionalized MBGS samples were not affected, in contrast to the conventional MBG (Fig. S3). These observations demonstrate that the post-grafting process is an effective method to graft amino or carboxylic functional groups onto MBGSs without negative effects.

It is necessary to evaluate the effects of implanted biomaterials on cell performance. As expected, both the MBGS and the functionalized MBGS samples presented comparable effects on the proliferation of bMSCs with the control, and the N-MBGS exhibited the most accelerated proliferation of bMSCs among the three samples. Furthermore, real-time PCR results showed that all three samples could improve the bone-relative gene expression of Runx2, ALP, BSP and OCN; the N-MBGS exhibited the most potent activity. The reasons for the behaviour of these MBGS samples can be explained as follows. 1) The release of Ca and Si ions from the MBGS samples could have improved cell proliferation and differentiation^6^. 2) The hydrophilic-hydrophobic balance of biomaterial surface is vital for cell adhesion. The hydrophilicity of cell membranes shows that hydrophilic surfaces of biomaterials are beneficial to cell adhesion. However, cells not only directly adhere to the surface of materials but also adhere to the materials through adhesive proteins, which are adsorbed on the surface of biomaterials. Excessive hydrophilicity of surfaces has an adverse effect on the adsorption of proteins, which can correspondingly influence cell adhesion^23^. Research shows that the adsorption capacity of fibronectin (FN) is weak on surfaces containing -OH or -COOH but is strong on surfaces containing -NH_2_^26^. Although -NH_2_ is a hydrophilic group, the hydrophilicity of -NH_2_ is lower than those of -OH and –COOH groups. Consequently, surfaces containing -NH_2_ exhibit hydrophilic-hydrophobic balance, which is beneficial for cell adhesion. 3) The capability of biomaterials to adsorb proteins on their surface can affect cell adhesion. In addition to the hydrophilic-hydrophobic balance of surfaces, the charge of these surfaces can also influence the adsorption of proteins. It is well known that most proteins possess low isoelectric points (PIs), which induces negatively charged proteins to adsorb onto the positively charged surface of an N-MBGS. Subsequently, cell adhesion that is achieved through the interaction of surfaces with these adhesive proteins is improved^24^. 4) As cell membranes possess negative charges on the outer surfaces, the surface of an N-MBGS containing positively charged -NH_2_ groups could further accelerate cell adhesion, proliferation and differentiation^27^,^28^,^29^,^40^.

It has been reported that even a 2.5 mm femur cavity defect could not autonomously heal after 8 weeks^41^. Consistent with the *in vitro* results, the *in vivo* experiments using a rabbit femur defect model showed that all the MBGS samples could promote the growth of new bone tissue (Fig. 7a). Quantitatively, the group implanted with the N-MBGS showed the largest new bone area of 38% ± 5.26%, while the C-MBGS and MBGS groups showed increased bone areas by 20.37% ± 5.01% and 12% ± 3.74%, respectively (Fig. 7B). Obviously, in addition to the surface chemistry of these scaffolds, the degradation rates of the implanted materials play a vital role in the process of new bone tissue growth. As observed in Fig. 7a, the implanted N-MBGS or C-MBGS degraded with the formation of new bone. However, most of the MBGS disappeared before the complete formation of new bone tissue, showing that the scaffold degraded too fast to support the growth of new bone.

**Figure 8:**
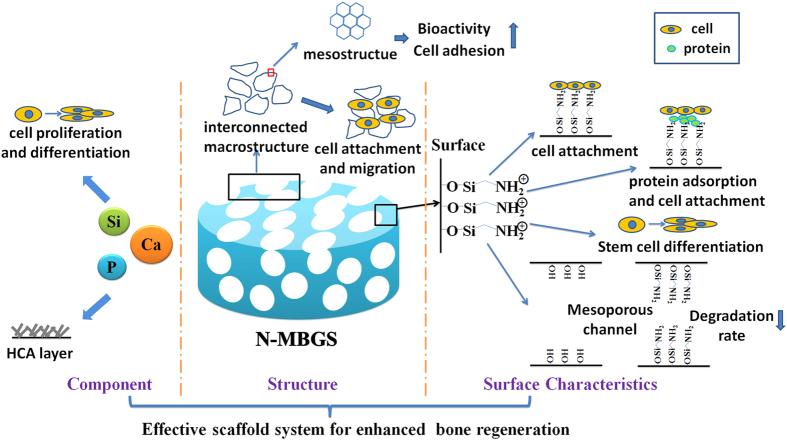
Schematic diagram of the N-MBGS as an effective scaffold for enhanced bone regeneration.

As discussed, Ca and Si ions released from the N-MBGS can improve cell proliferation and differentiation, while Ca and P ions can accelerate the formation of the HCA layer. The interconnected macroporous network of an N-MBGS is helpful for cell adhesion and migration, and in particular, the mesopores enhance the bioactivity and further promote cell adhesion. Surface modifications with amino groups not only provide suitable surfaces for cell to adhere, proliferate and differentiate but also decrease degradation rates to adapt new bone formation. As a result, the novel components, structure and surface characteristics endow N-MBGS with excellent osteogenetic performances *in vitro* and *in vivo* (Fig. 8).

## Methods

### Preparation of MBG powder

MBG 80S15C (S and C represent SiO_2_ and CaO, respectively; 80 and 15 are the molar ratios of SiO_2_ and CaO in percentages, respectively) was demonstrated to possess the best bioactivity and was prepared following a reported procedure for evaporation-induced self-assembly (EISA)^2^. In a typical synthesis, 4.0 g of P123 (Mw = 5800), 6.7 g of tetraethyl orthosilicate (TEOS, 98%), 1.4 g of Ca(NO_3_)_2_·4H_2_O, 0.73 g of triethyl phosphate (TEP, 99.8%) and 1.0 g of 0.5 M HCl were dissolved in 60 g of ethanol (Si/Ca/P = 80:15:5, molar ratio) and stirred at room temperature for 1 d. Afterwards, the resulting solution was transferred to a Petri dish to undergo the EISA process. The dried gel was calcined at 700 °C for 5 h and subsequently fully ground to obtain the MBG 80S15C powder.

### Preparation of MBG scaffold

MBG scaffolds (MBGSs) were prepared using a simple powder processing technique. Polyvinylpyrrolidone (PVP, K30) and polyethyleneglycol (PEG, Mw = 6000) particles were used as a binder and porogen, respectively. Briefly, MBG 80S15C powder, PVP and PEG particles (MBG:PVP:PEG = 1:0.2:1, weight ratio) were mixed well and transferred into a cylindrical mold with a diameter of 15 mm. The mold containing the mixture was pressed at 5 MPa to obtain a cylindrical green body with a diameter of 15 mm. Subsequently, the green body was calcined at 600 °C (ramp of 1 °C/min) for 5 h to obtain the MBGS. MBGSs with different sizes and shapes were prepared by using various moulds.

### Preparation of functionalized MBGSs

3-Aminopropyltrimethoxysilane (APTMS, 97%) and triethoxysilylpropyl succinic anhydride (TESPSA, 94%) were used to graft amino or carboxylic functional groups onto the MBGS, respectively. The preparation process is described as follows: MBGS was refluxed in 50 ml of dry toluene containing a certain amount of APTMS or TESPSA (APTMS/TESPSA:SiO_2_ = 0.3:1, molar ratio) at 80 °C for 12 h. After cooling to room temperature, the MBGS was collected by filtration, washed with toluene and distilled water and then dried under a vacuum at 80 °C to obtain the MBGSs functionalized with amino or carboxylic groups.

### Characterization of the MBGS and functionalized MBGSs

The morphologies, macroporous structures and sizes of the MBGSs were observed by digital microscopy (AM4112PT, Dino-Lite, Taiwan). The microstructures of the macroporous walls were investigated by scanning electron microscopy (SEM, Hitachi S4800 electron microscope). The ordered mesoporous structures of the scaffolds were confirmed by small-angle X-ray diffraction (SAXRD, Bruker D8 Focus diffractometer). Brunauere-Emmette-Teller (BET) and Barrete-Joynere-Halenda (BJH) analyses were conducted to determine the specific surface areas, pore volumes and pore size distributions of the MBGSs using a surface area and pore size analyser (Quantachrome NOVA 4200e, USA). FT-IR spectra were obtained on a Nicolet 5700 Thermo FT-IR spectrometer to determine if the functional groups were successfully grafted to the scaffolds. Macroporous diameters were measured using mercury porosimetry (AutoPore IV 950).

The compressive strength of the MBGS and functionalized MBGSs (10 × 10 × 10 mm) were tested using a computer controlled universal testing machine (Instron 5592, Instron Wolpert, Darmstadt, Germany) at a cross-head speed of 0.5 mm/min. Five samples were used as replicates for this experiment.

The porosities of the MBGS and functionalized MBGSs were measured using Archimedes’ principle according to previous publications^33^: scaffolds with a size of 10 × 10 × 10 mm were used for the measurement and water was used as the liquid medium. Porosity (P) was calculated according to the following equation: P = (W_2_-W_1_)/(W_2_-W_3_) × 100%, where W_1_ is the dry weight of the scaffolds, W_2_ is the weight of the scaffolds saturated with water, and W_3_ is the weight of the scaffolds suspended in water.

### Ion release, degradation and bioactivity of the MBGS and functionalized MBGSs *in vitro*

To investigate the extents of ions release of the scaffolds, simulated body fluids (SBF) were prepared according to Kokubo^42^. Scaffolds were soaked in SBF at 37 °C for 0, 2, 8, 16, 24, 48 and 72 h, and the ratio of the solution volume to the scaffold mass was set to 200 mL/g. The concentrations of Ca, P and Si ions in the SBF were determined with inductive coupled plasma atomic emission spectrometry (ICP-AES, Perkin-Elmer Optima 7000DV).

The degradation of scaffolds was evaluated by measuring Si ion concentrations in PBS (pH = 7.4)^33^. The percentage of weight loss was calculated as follows: weight loss (%) = (*C*_Si_ × *V*_Si_)/*M*_Si_, where *C*_Si_, *V*_Si_, and *M*_Si_ represent the Si concentration in PBS, volume of PBS (mL) and Si content (mg) of the samples, respectively.

To investigate the *in vitro* bioactivity of the scaffolds, they were soaked in SBF at 37 °C for 24 h, and apatite formation of the scaffolds was determined by wide-angle X-ray diffraction (WAXRD, Bruker D8 Focus diffractometer) and FT-IR spectrometry.

### Isolation and culture of rabbit bone marrow stromal cells (bMSCs)

Under general anaesthesia with intramuscular ketamine (10 mg/kg) and xylazine (3 mg/kg), bone marrow was harvested from the fibula of rabbits and bMSCs were cultured in Dulbecco’s modified Eagle’s medium (DMEM, Gibco) with 10% foetal bovine serum (FBS, Gibco) in a humidified incubator at 37 °C and 5% CO_2_, as described previously^43^,^44^. The cells were cultured for approximately 10 days until a confluence of approximately 80% was reached following the first passage; the medium was then replaced with osteogenic medium (DMEM, 10% FBS, 50 mg/mL L-ascorbic acid, 10 mM glycerophosphate and 100 nM dexamethasone). It usually took 6–7 days for an additional passage to occur; bMSCs obtained after the second passage were used for further studies. All animal procedures in the present study were approved by the Animal Research Committee of the Ninth People’s Hospital affiliated with Shanghai Jiao Tong University’s School of Medicine, and the methods were conducted in accordance with approved guidelines and regulations.

### Cell adhesion and proliferation assay

The adhesion and spreading of bMSCs cultured on the different samples were examined after 24 h. After 24 h of culture, scaffolds with bMSCs were washed with PBS twice, fixed with 3.7% formaldehyde, and then dehydrated in ascending concentrations of ethanol (30, 50, 70, 90, 95 and 100 (v/v)) for 5 min. The scaffolds were dried, and cell morphologies were observed using SEM. The MTT assay was used to assess the number of viable cells. In brief, bMSCs were seeded on the MBGS, N-MBGS and C-MBGS in 24-well plates in triplicate, cultured from 1 day to 5 days in medium containing MTT and incubated for 4 h at 37 °C. Dimethylsulfoxide (DMSO, Sigma, USA) was used to stop the reaction, and the absorbance value was measured at 490 nm with a microplate reader (Bio-Tek, Elx800). The results are expressed as units of optical density (O.D.) absorbance values. All experiments were performed in triplicate.

### Cell osteogenic differentiation assay

The osteogenic differentiation capabilities of bMSCs cultured on the scaffolds were evaluated by determining ALP activity and the expression of osteogenic genes. To determine the early differentiation of bMSCs stimulated by MBG, cells (8 × 10^4^ cells/well) were seeded on the MBGS and functionalized MBGSs. After 7 days of osteogenic induction, ALP staining was carried out using an ALP staining kit (Beyotime, Shanghai, China). Meanwhile, ALP activity was determined by utilizing p-nitrophenyl phosphate (pNPP) as a substrate after 7 days. The ALP activity of cells lysed with 0.1% Triton 100 in 10 mM Tris HCl was quantified by absorbance measurements at 405 nm. Total protein content was determined with the Bradford method using a Bio-Rad protein assay kit (Bio-Rad, Richmond, CA, USA). The ALP levels were normalized to the total protein content at the end of the experiment.

A real-time polymerase chain reaction (PCR) assay was performed to study the osteogenic gene expression of bMSCs seeded on the MBGS and functionalized MBGSs. Briefly, total RNA was harvested using TRIzol reagent (Invitrogen, USA) at 7 days^45^. The RNA was used to synthesize complementary DNA (cDNA) with a PrimeScript 1st Strand cDNA Synthesis kit (TaKaRa, Japan). Real-time PCR analysis was performed using a Bio-Rad real-time PCR system (Bio-Rad, USA) on markers of runt-related transcription factor 2 (Runx2), alkaline phosphatase (ALP), bone sialoprotein (BSP), and osteocalcin (OCN). Primer sequences for Runx2, ALP, BSP, OCN and GAPDH are listed in Table S1. All experiments were performed in triplicate.

### Surgical procedure

Twelve-week-old female New Zealand white rabbits (wt. 2 kg) were obtained from the Ninth People’s Hospital Animal Center (Shanghai, China). Eighteen female New Zealand rabbits were divided into three groups (i.e., MBGS, N-MBGS and C-MBGS, Φ5 mm*3 mm)^34^. Briefly, the animals were anaesthetized by intramuscular injection of sodium pentobarbital (0.02 g/kg) under rigorous aseptic conditions. A 1.0 to 1.5 cm sagittal incision was made on the shaved legs, and the femurs were exposed by blunt dissection. Ø 5 mm defects were created by trephine bur (Fine Science Tools, Foster City, CA, USA). After the defects were washed with physiological saline, the MBGS, N-MBGS and C-MBGS were implanted (n = 6 rabbit/batch) and the incisions were closed in layers using 4–0 resorbable sutures.

A polychrome sequential labelling method was used to label the mineralized tissue and assess the time course of new bone formation and mineralization. At 4 and 8 weeks after surgery, the animals were intraperitoneally administered with 30 mg/kg of alizarin red (AL, Sigma) and 20 mg/kg of calcein (CA, Sigma). All procedures were approved by the Ninth People’s Hospital affiliated with Shanghai Jiao Tong University’s Committee on the Use and Care of Animals, and the methods were carried out in accordance with approved guidelines and regulations.

### Micro-CT analysis

At 12 weeks post-operation, all the rabbits were sacrificed, and specimens were harvested. The morphologies of the reconstructed skulls were assessed using an animal micro-CT scanner (eXplore Locus, GE Healthcare Biosciences, London, UK). Briefly, the specimens were scanned with parameters including an X-ray tube potential of 80 kV, a tube current of 0.45 mA, and 15-μm voxel resolution, as previously described. After the micro-CT scans, the bones were visualized using software with three-dimensional isosurface renderings. Micro-CT measurements included the quantification of bone volume (BV) in the bone defects.

### Histological evaluation

The extracted femora, with the soft tissue cleaned, were fixed in 10% neutral buffered formaldehyde, dehydrated in ascending concentrations of ethyl alcohol ranging from 70% to 100% and finally embedded in polymethylmethacrylate. The specimens were cut into 200-μm thick sections using a microtome (Leica) and were subsequently ground and polished to a thickness of 40–50 μm. To observe fluorescent labelling of the sections, a confocal laser scanning microscope (Leica TCS Sp2 AOBS) was used. Excitation/emission wavelengths for each of the fluorochromes were 543/617 nm (AL, red) and 488/517 nm (CA, green). Four slides were randomly selected from serial sections of each sample and were used to analyse the percentages of newly formed bone area in the raised areas. Briefly, the decalcified sections were stained with Van Gieson’s picro fuchsin for histological observation. The percentage of newly formed bone area for each group in the raised area at week 12 was calculated using an automated image analysis system (Image-Pro Plus).

### Statistical analysis

All numerical data were expressed as the mean ± standard deviation. Statistical analyses were performed by ANOVA and SNK post hoc tests based on normal distributions and equal variance assumption tests. All statistical analyses were performed using the SAS 8.2 statistical software package (SAS), and significant differences were indicated by p < 0.05.

## Additional Information

**How to cite this article**: Zhang, X. *et al.* Functionalized mesoporous bioactive glass scaffolds for enhanced bone tissue regeneration. *Sci. Rep.* **6**, 19361; doi: 10.1038/srep19361 (2016).

## Supplementary Material

Supplementary Information

## Figures and Tables

**Table 1:** The compressive strength and porosity of samples.

Samples	Compressive strength (MPa)	Porosity (%)
MBGS	0.30 ± 0.03	73 ± 2
N-MBGS	0.34 ± 0.02	68 ± 2
C-MBGS	0.35 ± 0.02	67 ± 2
Control*	0.06	86

^*^MBG scaffolds prepared by the polyurethane foam template method^33^.

**Table 2:** Mesoporous structure parameters of samples.

Samples	S_BET_ (m^2^/g)*	V_pore_ (cc/g)**	D_p_ (nm)***
MBG	352.1	0.44	4.63
MBGS	332.9	0.42	4.63
N-MBGS	182.1	0.21	4.04
C-MBGS	193.8	0.20	4.06

^*^Specific surface area determined by BET method.

^**^Pore volume calculated in peak of pressure.

^***^Pore size determined by BJH method (the adsorption branch).
