# Supplementary material for: Functionalized mesoporous bioactive glass scaffolds for enhanced bone tissue regeneration
Source: Sci Rep. 2016 Jan 14;6:19361. doi: 10.1038/srep19361 (PMC4725866; doi:10.1038/srep19361)
Supplement: Supplementary Information [file srep19361-s1.pdf]

Functionalized mesoporous bioactive glass scaffolds for enhanced bone tissue regeneration

Xingdi Zhang[1, +], Deliang Zeng[2, +], Nan Li[1], Jin Wen[2], Xinquan Jiang[2*], Changsheng Liu[3] and Yongsheng Li[1*]

1. Lab of Low-Dimensional Materials Chemistry, Key Laboratory for Ultrafine Materials of Ministry of Education, School of Materials Science and Engineering, East China University of Science and Technology, Shanghai 200237, China

2. Department of Prosthodontics, Ninth People's Hospital affiliated to Shanghai Jiao Tong University, School of Medicine, Shanghai 200011, China

3. Engineering Research Center for Biomedical Materials of Ministry of Education, East China University of Science and Technology, Shanghai 200237, China

***Corresponding authors. E-mail addresses: ysli@ecust.edu.cn (Y. Li), xinquanj@aliyun.cn (X. Jiang)**

[+] These authors contributed equally to this work.

Supplementary Information

Gene	Direction	Primer sequence (5′-3′)
Runx2	Forward	ATCCAGCCACCTTCACTTACACC
	Reverse	GGGACCATTGGGAACTGATAGG
ALP	Forward	GTCCCACAAGAGCCCACAAT
	Reverse	CAACGGCAGAGCCAGGAAT
BSP	Forward	TGGATGAACCAAGCGTGGA
	Reverse	TCGCCTGACTGTCGATAGCA
OCN	Forward	CAGTAAGGTGGTGAATAGACTCCG
	Reverse	GGTGCCATAGATGCGCTTG
GAPDH	Forward	TTCGACAGTCAGCCGCATCTT
	Reverse	ATCCGTTGACTCCGACCTTCA

Table S1 Nucleotide sequences for real-time RT-PCR primers

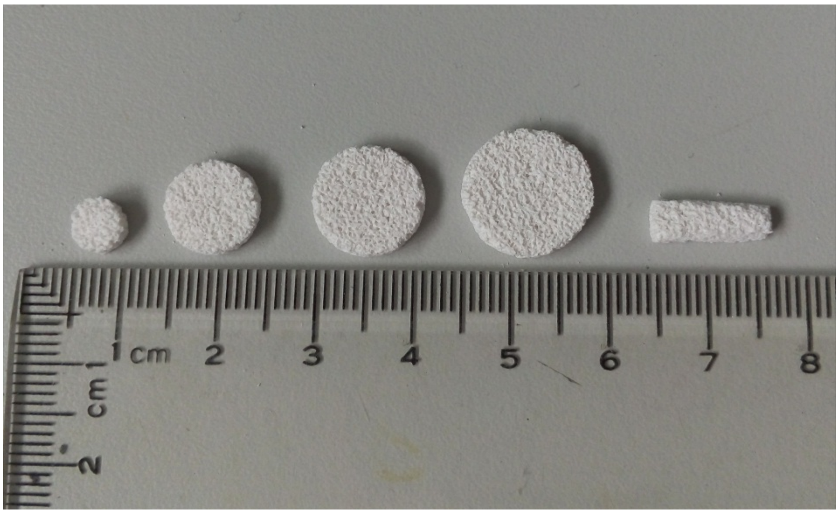

Figure S1. Photograph of MBGS with different sizes or shapes

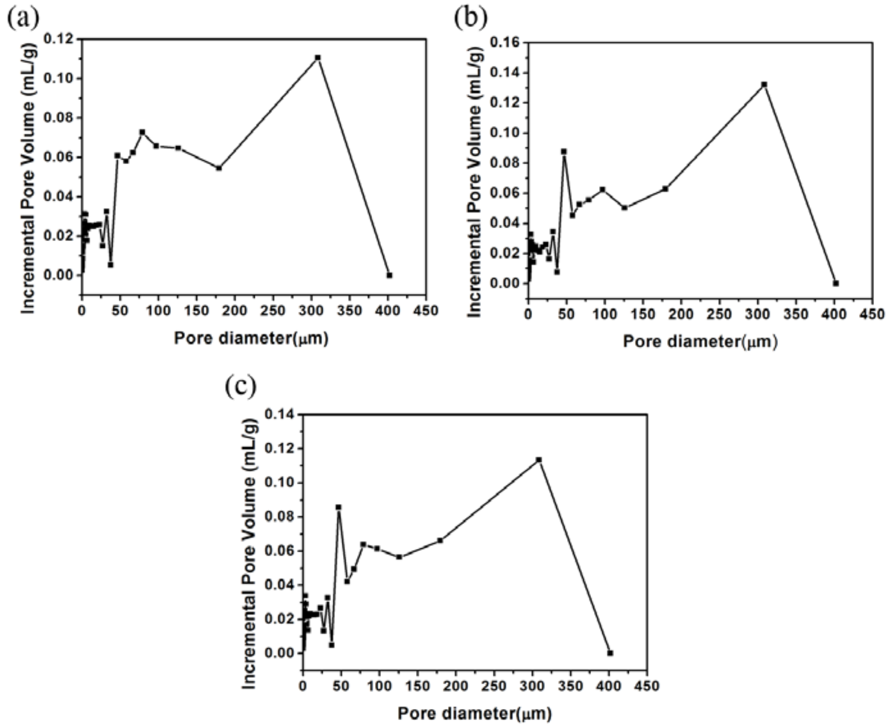

Figure S2. Mean macroporous diameter of (a) MBGS, (B) N-MBGS and (c) C-MBGS measured by mercury intrusion porosimetry(MIP) method

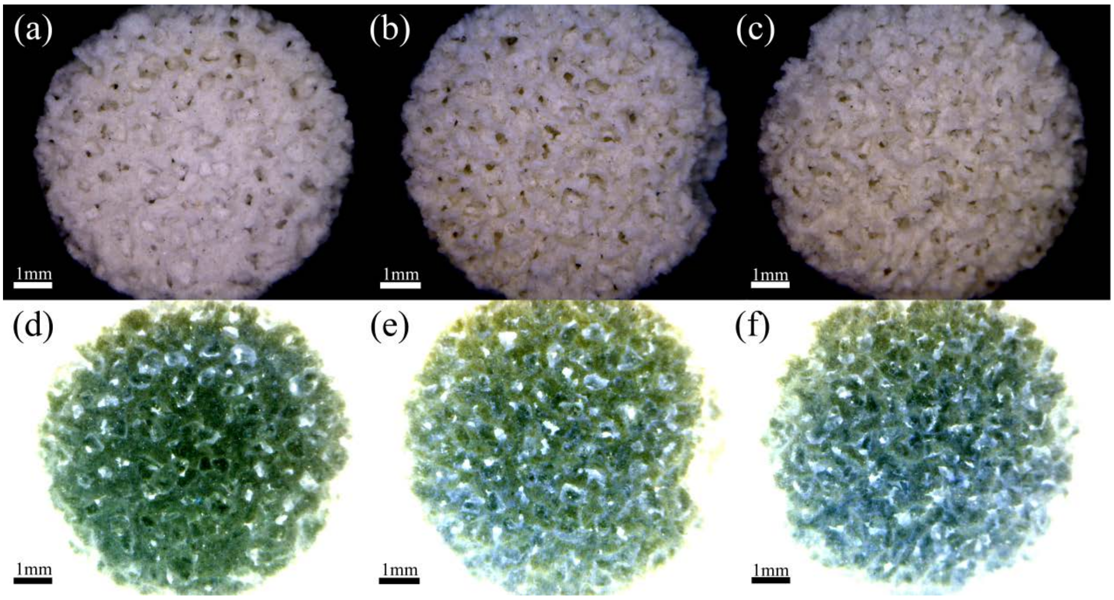

Figure S3. Digital microscopic photograph of (a) MBGS, (b) N-MBGS and (c) C-MBGS; Reverse color photographs of (d)MBGS, (e) N-MBGS and (f) C-MBGS.

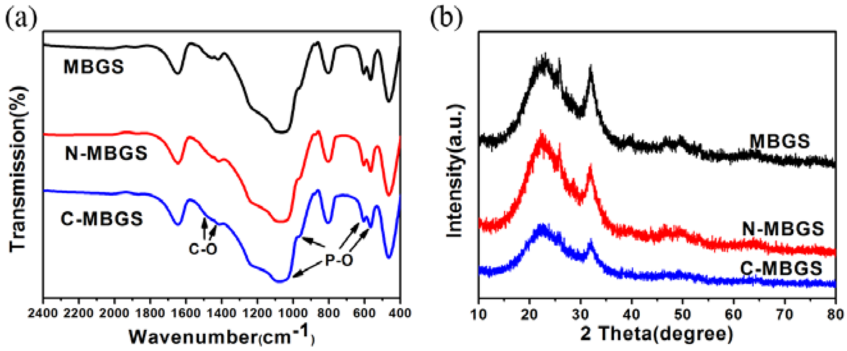

Figure S4. (a) FT-IR spectra and (b) Wide-angle XRD patterns of MBGS, N-MBGS and C-MBGS after soaking in simulated body fluids for 24h

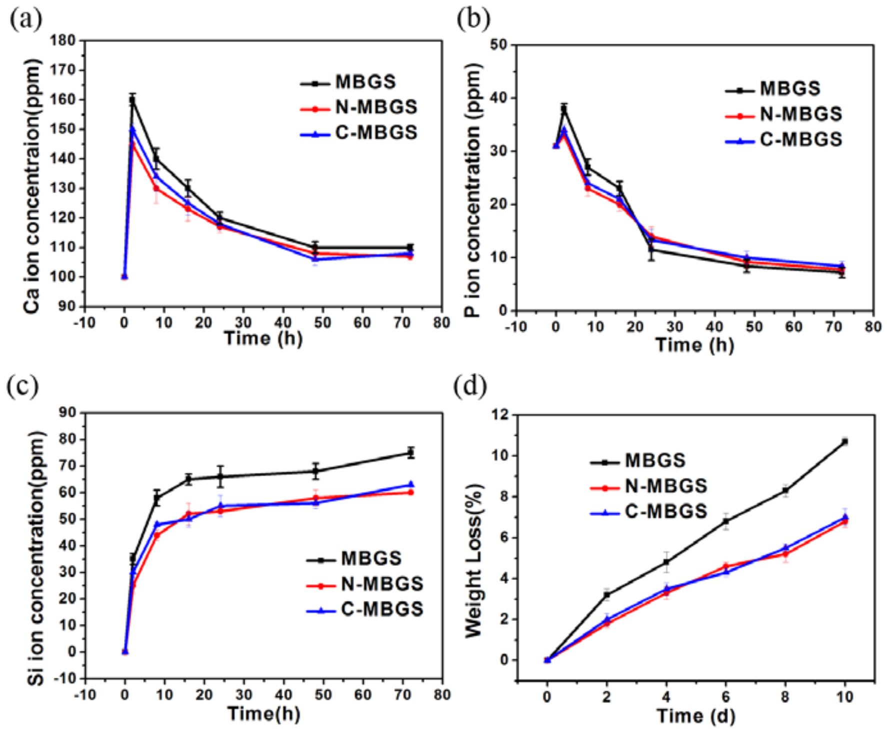

Figure S5. The change of (a) Ca, (b) P and (c) Si ions in SBF after soaking MBGS, N-MBGS and C-MBGS for different times. (d) Weight loss of scaffolds in PBS (pH=7.4).
